# Blood Plasma Metabolic Profile of Newborns with Hypoxic-Ischaemic Encephalopathy by GC-MS

**DOI:** 10.1155/2021/6677271

**Published:** 2021-06-24

**Authors:** Yanjuan Jia, Xiaoni Jia, Hui Xu, Lan Gao, Chaojun Wei, Yonghong Li, Xia Liu, Xiaoling Gao, Li Wei

**Affiliations:** ^1^NHC Key Laboratory of Diagnosis and Therapy of Gastrointestinal Tumor, Gansu Provincial Hospital, Lanzhou, China 730000; ^2^The Institute of Clinical Research and Translational Medicine, Gansu Provincial Hospital, Lanzhou, China 730000; ^3^The Neonatal Department, Qingyang People's Hospital, Qingyang, China 73000; ^4^The Department of Life Science, Lanzhou University, Lanzhou, China 730000; ^5^The Laboratory Center, Gansu Provincial Hospital, Lanzhou, China 730000

## Abstract

**Background:**

Early diagnosis of hypoxic-ischaemic encephalopathy (HIE) is crucial in preventing neurodevelopmental disabilities and reducing morbidity and mortality. The study was to investigate the plasma metabolic signatures in the peripheral blood of HIE newborns and explore the potential diagnostic biomarkers.

**Method:**

In the present study, 24 newborns with HIE and 24 healthy controls were recruited. The plasma metabolites were measured by gas chromatography-mass spectrometry (GC-MS), and the raw data was standardized by the EigenMS method. Significantly differential metabolites were identified by multivariate statistics. Pathway enrichment was performed by bioinformatics analysis. Meanwhile, the diagnostic value of candidate biomarkers was evaluated.

**Result:**

The multivariate statistical models showed a robust capacity to distinguish the HIE cases from the controls. 52 metabolites were completely annotated. 331 significantly changed pathways were enriched based on seven databases, including 33 overlapped pathways. Most of them were related to amino acid metabolism, energy metabolism, neurotransmitter biosynthesis, pyrimidine metabolism, the regulation of HIF by oxygen, and GPCR downstream signaling. 14 candidate metabolites showed great diagnostic potential on HIE. Among them, alpha-ketoglutaric acid has the potential to assess the severity of HIE in particular.

**Conclusion:**

The blood plasma metabolic profile could comprehensively reflect the metabolic disorders of the whole body under hypoxia-ischaemic injury. Several candidate metabolites may serve as promising biomarkers for the early diagnosis of HIE. Further validation based on large clinical samples and the establishment of guidelines for the clinical application of mass spectrometry data standardization methods are imperative in the future.

## 1. Introduction

Hypoxic-ischaemic encephalopathy (HIE) is mainly caused by perinatal hypoxia-ischaemia, being responsible for nearly 23% of neonatal deaths worldwide [1, 2]. The incidence of HIE is 1.5 out of 1000 live births annually, which is higher in developing countries [3]. Approximately 25% to 30% of survivors may be at risk of lifelong neurological impairment and disabilities [4]. The efficiency of many current existing methods on HIE diagnosis, such as Apgar series score, cord blood gas, cranial ultrasound, and magnetic resonance imaging (MRI), have proven to be controversial and modest due to individual subjectivity, methodological shortcomings, and the complexity of the encephalopathy [5–12]. For instance, MRI, as one of the main popular diagnostic tools, often shows false-negative results due to its low sensitivity [13]. What is worse, unstable newborns may not be able to tolerate the transport or the duration of brain MRI scanning [14]. Indeed, no fast and reliable biomarker is currently available to help clinicians accurately assess the degree of the hypoxic-ischaemic (HI) injury [15].

In recent years, metabolomics is widely used to screen biomarkers for early diagnosis of disease and pathogenesis research [16]. The metabolic profiles have been established in biological fluid or tissue of several animal models for HIE and umbilical cord blood (UCB) obtained from HIE infants [17]. Several altered metabolites and perturbed metabolic pathways have been identified that may provide some information for establishing a panel of biomarkers for the early diagnosis of HIE. Actually, HI injury could seriously affect the integrity and permeability of the blood-brain barrier [18].Thus, various small molecules, including some metabolites, may be secreted into the peripheral circulation, and detection of the metabolic profile may play a vital role in the comprehensive understanding of HIE. So far, the blood plasma metabolic profile for HIE newborns compared with that for the normal remains unclear. Herein, we aimed to systematically analyze the plasma metabolic changes in the plasma of HIE newborns using metabolomics to explore a set of noninvasive biomarkers for the early diagnosis of HIE and early neuroprotection treatment.

## 2. Materials and Methods

### 2.1. Ethics Approval and Consent to Participate

The study was conducted according to the Declaration of Helsinki and approved by the Ethics Committee of the Gansu Provincial Hospital and Qingyang People's Hospital (Gansu, China). The informed consent forms were signed by all included newborns' parents before admission, agreeing on the use of clinical test data and the rest of human biosamples for scientific research by the hospital. The blood samples used in the study were the rest of the sample after the blood routine tests or the measurement of ABO blood type.

### 2.2. Study Design

76 newborn infants were recruited between November 2017 and March 2019. 28 were excluded because of sample hemolysis, severe jaundice, or insufficient quantity. 48 infants were left for the study. The HIE cases were enrolled from the Neonatal Intensive Care Unit of Qingyang People's Hospital. Newborns with neural tube defects, alcohol and drug embryopathies, serious cardiac abnormality hydrops, important gastrointestinal malformation, congenital malformations, and hereditary metabolic diseases were excluded. Inclusion criteria for HIE group were as follows: (1) there were severe signs of fetal distress or an abnormal obstetric history related to fetal distress; (2) gestational age ≥ 35 weeks, birth weight ≥ 1800 g; (3) 5 min Apgar score ≤ 7 [8]; and (4) obvious nervous system symptom. Meanwhile, magnetic resonance imaging (MRI) was used to separate the severity of HIE [11, 12]. The injury was scored by using the modified Barkovich score based on the basal ganglia and thalami in combination with cortical involvement, the watershed areas, and the posterior limb of the internal capsule [19–22]. There were 14 mild HIE and 10 moderate/severe HIE included in the study. The control group was enrolled from the Obstetrics Department of Qingyang People's Hospital. Inclusion criteria for the control group were as follows: (1) gestational age ≥ 35 weeks, birth weight ≥ 1800 g; (2) 5 min Apgar score ≥ 9. All cases had demographic and clinical details collected.

### 2.3. Sample Collection and Storage

2 mL blood was drawn from the femoral or radial artery into the EDTA-K_2_ tube after birth. Then, the samples were transported to the laboratory center of Qingyang People's Hospital for a blood routine test by well-trained professionals within 20 minutes. Next, the samples were processed according to the following standard operating procedure: samples were centrifuged at 3000 × g at room temperature for 10 minutes, and then, two aliquots of 200 *μ*L of plasma were transferred to new microtubes and stored at -80°C. All samples were transported to the Gansu Provincial Hospital for mass spectrometry detection using dry ice.

### 2.4. Sample Extraction

50 *μ*L plasma was taken into the 1.5 mL Eppendorf (EP) tubes, extracted with 200 *μ*L methanol, added 5 *μ*L of adonitol (0.5 mg/mL stock in dH_2_O) as internal standard, vortex mixing for the 30 s, and treated with ultrasound for 10 min (incubated in ice water), then centrifuged for 15 min at 12000 rpm, 4°C. After that, 180 *μ*L supernatant of each sample was transferred into fresh 1.5 mL EP tubes, and 40 *μ*L from each sample was taken into fresh 1.5 mL EP tubes as a quality control (QC) sample. Furthermore, the extracts were dried completely in a vacuum concentrator without heating, then 30 *μ*L methoxyamination hydrochloride (20 mg/mL in pyridine) incubated for 30 min at 80°C was added, and 40 *μ*L of the BSTFA regent (1% TMCS, *v*/*v*) to the sample aliquots was added, incubated for 1.5 h at 70°C. 5 *μ*L of FAMEs (standard mixture of fatty acid methyl esters, C8-C30, 1 mg/mL, in chloroform) was added to the QC sample when cooling to the room temperature. All samples were analyzed by the gas chromatograph system coupled with a Pegasus HT time-of-flight mass spectrometer (GC-TOF-MS).

### 2.5. GC-MS Analysis

GC-TOF-MS analysis was performed using an Agilent 7890 gas chromatograph system equipped with a Pegasus HT time-of-flight mass spectrometer. The system has a DB-5MS capillary column coated with 5% diphenyl cross-linked with 95% dimethylpolysiloxane (30 m × 250 *μ*m inner diameter, 0.25 *μ*m film thickness; J&W Scientific, Folsom, CA, USA). A 1 *μ*L aliquot of the analyte was injected in the splitless mode. Helium was used as the carrier gas with a purge flow rate of 3 mL min^−1^ at the front inlet and a gas flow rate of 1 mL min^−1^ through the column. The initial temperature was kept at 50°C for 1 min, then raised to 310°C at a rate of 20°C min^−1^, then kept for 6 min at 310°C. The temperature of injection, transfer line, and ion source were 280, 280, and 250°C, respectively. The energy in the electron impact mode was -70 eV. The mass spectrometry data were obtained in the full-scan mode with the m/z range of 50-500 at a rate of 12.5 spectra per second after a solvent delay of 4.83 min.

### 2.6. Data Processing and Normalization

The raw peak exaction, data baseline filtering and baseline calibration, peak alignment, deconvolution analysis, peak identification, and peak area integration were carried out by using Chroma TOF 4.3X software of LECO Corporation and the LECO-Fiehn Rtx5 database [23]. The annotation of mass spectral data is the first step for the data interpretation. Mass spectrum match and retention index match were considered simultaneously in metabolite identification. Peaks detected in <50% of QC samples or RSD > 30% in QC samples were removed [24]. The missing values were filled up by half of the minimum value. Referring to the method for the normalization of blood metabolomics data [25], the EigenMS method has been selected with higher entropy across groups for data normalization (Supplementary Table 1). The log2 transformation of the peak area and the normalization were performed on the MetaboGroupS platform (https://www.omicsolution.org/wukong/MetaboGroupS/) for further analysis.

### 2.7. Statistical Analysis and Pathway Enrichment

Student's test and Fisher's exact test were applied to analyze demographic and clinical data by SPSS version 22 (SPSS Inc., Chicago, IL, USA). Principal component analysis (PCA) was carried out to reduce the dimension of the data and visualize the distribution and the grouping of the samples. 95% confidence interval in the PCA score plot was used as the threshold to identify potential outliers in the dataset. Orthogonal partial least squares discriminant analysis (OPLS-DA) was applied to improve understanding of the variables responsible for the classification and obtain the contribution of each variable to the model. Both analysis approaches were conducted by SIMCA software (V15.0.2, Sartorius Stedim Data Analytics AB, Umea, Sweden). A 7-fold cross-validation was performed to check the robustness and predictive ability of the OPLS-DA model. The performance of the model was evaluated by the goodness of fit parameters *R*^2^ and *Q*^2^. A permutation test was performed to validate the OPLS-DA model by resampling the model 200 times under the random permutation. Significantly differential metabolites were identified based on the combination of the first principal component of variable importance in projection (VIP) values > 1.0 with a two-tailed Student's *t*-test (*p* < 0.05) between the control and HIE groups.

The biological functions and pathway analysis of significantly changed metabolites were enriched by the MetaboAnalyst 4.0 and the “Wu Kong” platform (https://www.omicsolution.org/wkomics/main/) based on several databases including the Kyoto Encyclopedia of Genes and Genomes (KEGG; http://www.genome.jp/kegg/), the PubChem Database (PCDB; https://www.ncbi.nlm.nih.gov/pccompound/), the Human Metabolome Database (HMDB; http://www.hmdb.ca/), and the Small Molecule Pathway Database (SMPDB; http://smpdb.ca/). The pathway enrichment analysis and pathway topology analysis of the differential metabolites were performed by Fisher's exact test and out-degree centrality. Visualization of hierarchical clustering and the correlation of the differential metabolites were conducted by R software (V3.6.1). The enrichment results were visualized by Cytoscape software (V 3.7.1). The receiver operating characteristic (ROC) was performed on the OmicShare platform (https://www.omicshare.com/tools/Home/Soft/roc).

## 3. Results

### 3.1. Study Population

The baseline clinical characteristics of the study population are presented in Table 1. There was no significant difference in neonatal sex, method of delivery, nationality, and maternal age between the HIE group and the control group. Gestational age, Apgar 1, Apgar 5, birth weight, leucocyte count, and lymphocyte count manifested a significant difference between the two groups (Table 1).

### 3.2. Identification of Differential Metabolites

**Figure 1:**
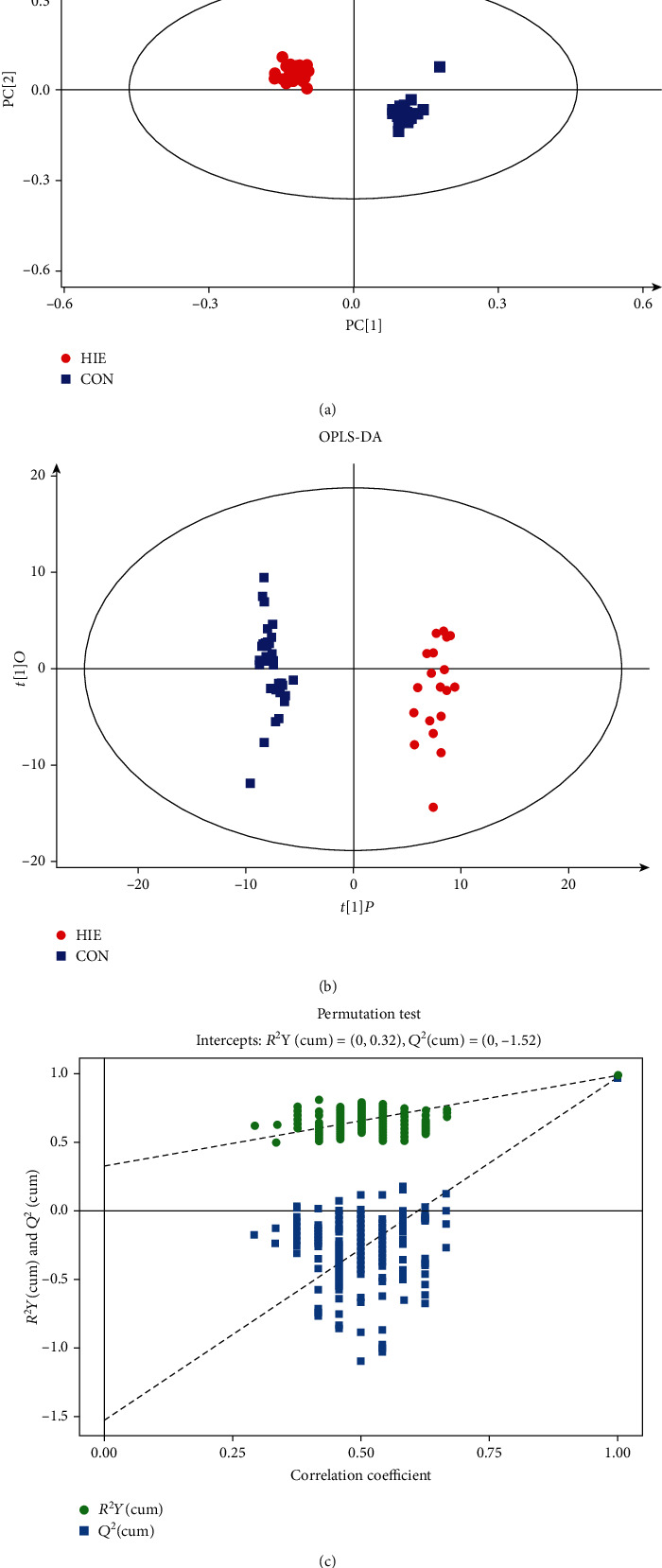
Multivariate statistical analysis on spectral data of neonatal plasma in groups HIE and CON: (a) PCA scattered plot; (b) OPLS-DA scattered plot; (c) permutation test. HIE: hypoxic-ischaemic encephalopathy; CON: healthy group.

The QC samples were distributed in the range of ±2std, suggesting the good stability of the system (Supplementary Figure 1). A total of 480 peaks were detected in both groups. After a series of data management including relative standard deviation denoising, missing values filled by half of the minimum value, and data normalization by the internal standard approach, finally, there were 343 peaks retained. Then, the EigenMS method was preferred to standardize the data based on the entropy across groups among the seven methods (Supplementary Table 1) [25]; the higher the entropy across groups, the better the model. The PCA score plot was made to examine the distribution of the HIE group and the control group (*R*^2^*Y* = 0.52, *Q*^2^ = 0.019; Figure 1(a)). The model showed a clear separation of the HIE group from the control group. OPLS-DA score plots further confirmed that this model was efficient and reliable. Goodness-of-fit parameters of the model were high, with an explanation capacity *R*^2^*Y* of 0.987 and a prediction capacity *Q*^2^ of 0.964 (maximum = 1) (Figure 1(b)).

**Figure 2:**
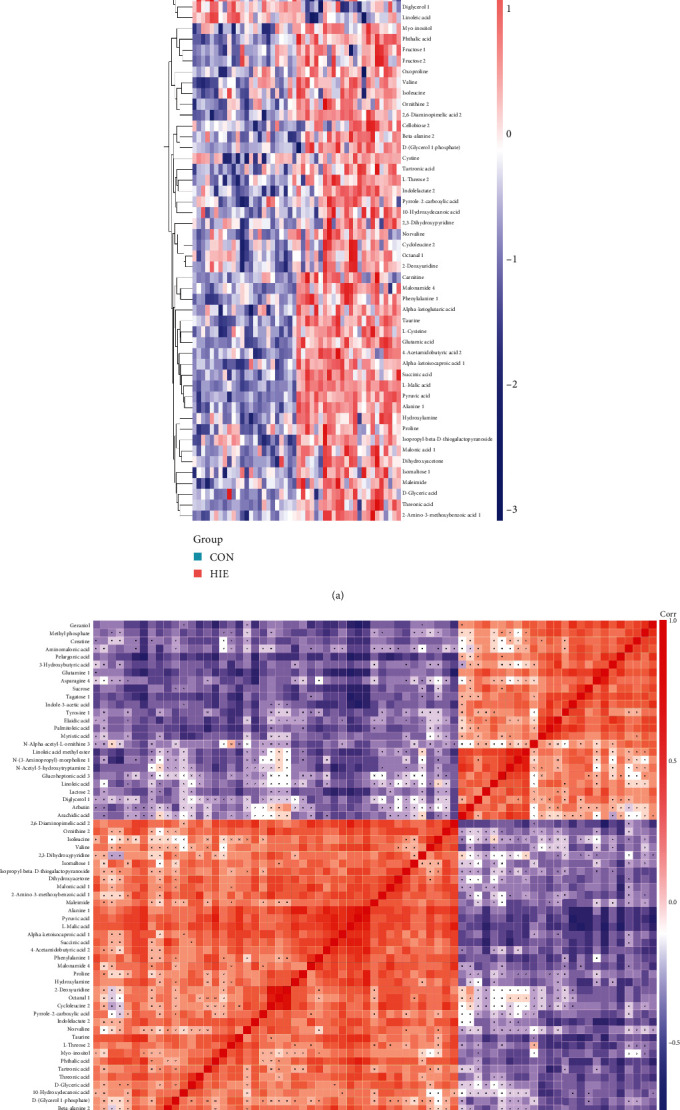
Heat map of hierarchical clustering and correlation analysis for group HIE vs. CON: (a) hierarchical clustering; (b) correlation analysis. Red indicates positive correlation and blue indicates negative correlation. The nonsignificant correlation was marked with fork number. HIE: hypoxic-ischaemic encephalopathy; CON: healthy group.

Moreover, the permutation tests showed that the *R*^2^*Y* and *Q*^2^ values from the constructed model (top right) were always higher than the simulated values of the permutation test (bottom left), suggesting that the OPLS-DA model was robust and not overfitted (Figure 1(c)). According to the VIP > 1.0 and *p* < 0.05, 71 differential metabolites were identified, of which 46 metabolites were upregulated and 25 were downregulated (Figure 2(a)). The correlation among the differential metabolites was calculated by Pearson analysis and was shown in Figure 2(b).

### 3.3. Annotation and Classification of Metabolites

The identified metabolites were annotated based on the databases of HMDB, KEGG, and PCDB. 52 differential metabolites were annotated out of a total of 71, including 33 upregulated metabolites and 19 downregulated metabolites (Supplementary Table 2). For their chemical properties, the metabolite subclasses of these potential biomarkers were grouped into the following categories: “amino acids, peptides, and analogs,” “carbohydrates and carbohydrate conjugates,” “fatty acids and conjugates,” “dicarboxylic acids and derivatives,” “benzoic acids and derivatives,” “beta hydroxy acids and derivatives,” and others (Supplementary Table 2).

### 3.4. Perturbed Metabolic Pathways in HIE

**Figure 3:**
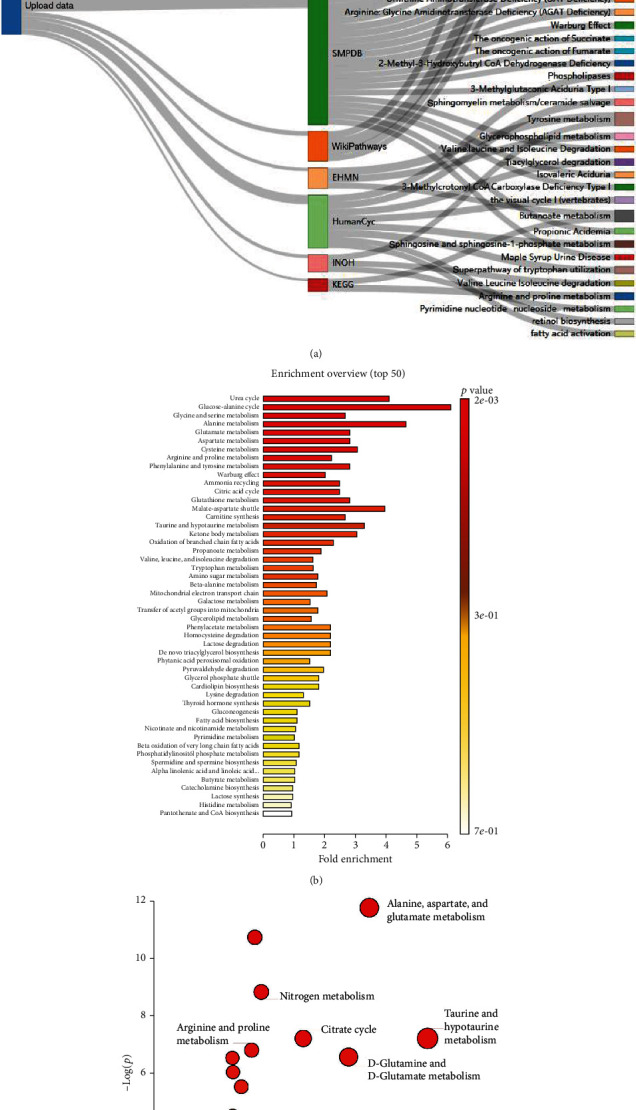
Pathway analysis of altered metabolites in neonatal plasma for group HIE vs. CON: (a) Sankey network based on Reactome, SMPDB, HMDB, WikiPathways, EHMN, HumanCyc, INOH, and KEGG; (b) barplot based on the SMPDB; (c) dot plot based on the KEGG. HIE: hypoxic-ischaemic encephalopathy; CON: healthy group.

**Figure 4:**
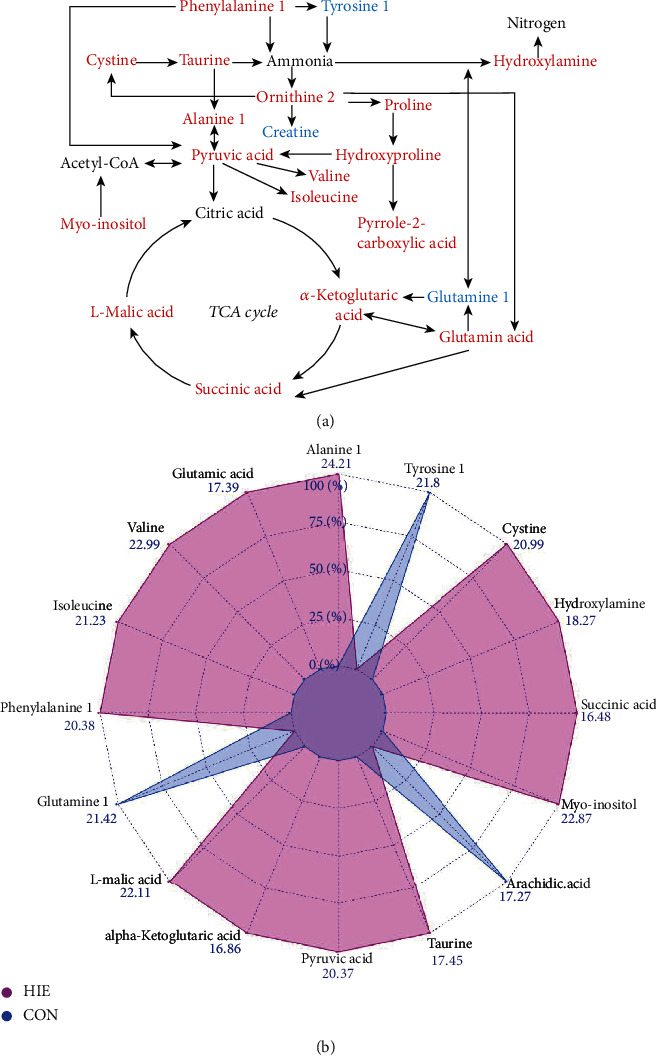
Assembly of the alterations of amino acid metabolic pathways in neonatal plasma for group HIE vs. CON. The colors correspond to the level of metabolites: red indicates increased levels, while blue indicates decreased levels. (a) Overview of the altered pathways of amino acid and energy metabolism; (b) radar chart comparing two groups on sixteen variables. TCA: tricarboxylic acid; HIE: hypoxic-ischaemic encephalopathy; CON: healthy group.

**Figure 5:**
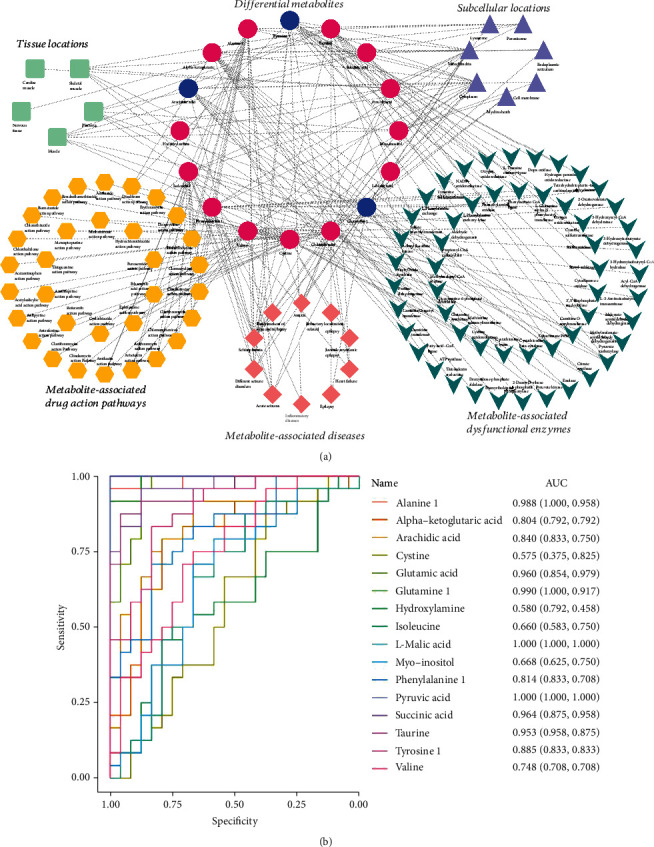
The enrichment analysis and prediction of diagnostic value of candidate differential metabolites. (a) Enrichment analysis of the differential metabolites. Five parts involved in the map including tissue locations, subcellular locations, metabolite-associated drug action pathways, metabolite-associated diseases, and metabolite-associated dysfunction enzymes. The same shape represented the same group. The colors correspond to the level of differential metabolites: red indicates increased levels, while blue indicates decreased levels. (b) Diagnostic value of 16 candidate differential metabolites. AUC: area under the curve; ROC: receiver operating characteristic.

Among the identified potential biomarkers, the metabolite pathway overrepresentation test was performed to comprehensively understand the biological process and altered pathways in HIE in comparison with the control group. The results showed that 331 pathways were significantly perturbed in HIE based on seven databases (*p* < 0.05, Supplementary Table 3), and the top 50 pathways were shown in the Sankey network (Figure 3(a)). Among them, 32 signaling pathways were jointly enriched in more than two databases (Supplementary Table 4), including GABA synthesis, release, reuptake and degradation, neurotransmitter uptake and metabolism in glial cells, pyrimidine metabolism, regulation of hypoxia-inducible factor (HIF) by oxygen, tricarboxylic acid (TCA) cycle, TCA cycle and respiratory electron transport, and tryptophan metabolism. Meanwhile, several pathways related to downregulated metabolites mainly included fatty acid biosynthesis, phospholipases, sphingomyelin metabolism, fatty acid activation, neurotransmitter release cycle, and free fatty acid receptors. Based on the SMPDB, the top 50 of enrichment results were shown in Figure 3(b). Based on the KEGG, significantly abnormal pathways including alanine, aspartate and glutamate metabolism, nitrogen metabolism, citrate cycle, taurine, and hypotaurine metabolism, arginine and proline metabolism, and D-glutamine and D-glutamate metabolism were marked in Figure 3(c). More than 37% of the differential metabolites were involved in the amino acid metabolism and energy metabolism and were reassembled into a brief plot (Figure 4(a)). Finally, integrating the results of pathway enrichment analysis with the published results on several animal models and UCB [26, 27], 16 metabolites were selected as a candidate biomarker set, including alanine, pyruvic acid, alpha-ketoglutaric acid, glutamic acid, succinic acid, phenylalanine1, cystine, valine, isoleucine, hydroxylamine, taurine, L-malic acid, and myo-inositol, glutamine, tyrosine, and arachidic acids. The relative expression levels of candidate biomarkers in both groups were shown in the radar plot (Figure 4(b)). Given the genome-scale network model of human metabolism, the tissue locations, subcellular locations, dysfunctional enzymes, drug action pathways, and diseases associated with these candidate biomarkers were enriched (Figure 5(a)). These blood metabolites were mainly located in the placenta, muscle, and myelin sheath (Figure 5(a)).

### 3.5. Diagnostic Capacity of the Candidate Metabolites

Through the logistic regression analysis, the diagnostic performance of each candidate biomarker was further evaluated (Figure 5(b)). The result showed that alanine 1, glutamic acid, glutamine 1, L-malic acid, succinic acid, pyruvic acid, and taurine showed an excellent performance with the area under the curve (AUC) more than 0.95 and with high specificity and sensitivity (>0.85). Arachidic acid, phenylalanine 1, alpha-ketoglutaric acid, and tyrosine 1 exhibited a good capacity (AUC > 0.80) and with specificity and sensitivity (>0.70). Valine, myo-inositol, and isoleucine showed a moderate capacity (AUC = 0.65‐0.75), suggesting that these metabolites could have potential diagnostic value for HIE except hydroxylamine and cystine (AUC = 0.58). Meanwhile, alpha-ketoglutaric acid and hydroxylamine had good diagnostic value in discriminating mild HIE from severe HIE (AUC > 0.70) (Supplementary Figure 2).

## 4. Discussions

HIE is characterized by depression of the level of consciousness, often accompanied by a series of complex clinical symptoms, such as abnormal muscle tone and power, respiratory depression, cerebral nerve dysfunction, and seizures. The metabolic profiles from animal models of HIE demonstrated the complex multifactorial nature of neonatal HIE [28–30]. Our study firstly reported the blood plasma metabolic profile of newborns with or without HIE. Significantly altered metabolites and the perturbed pathway have been found in HIE. And the plasma levels of some metabolites showed a potential role as a biomarker for the rapid diagnosis of HIE at an early stage.

### 4.1. Energy Metabolism and Amino Acid Metabolism

In our study, more than 1/3 of changed metabolites in HIE was related to mitochondrial energy metabolism and amino acid metabolism. Abnormalities in energy metabolism mainly involved changes in the intermediates of the TCA cycle and changes in the energy sources. In the TCA cycle, three intermediates including alpha-ketoglutaric acid, succinic acid, and L-malic acid were predominantly elevated in HIE, which was consistent with the results of urine metabolomics [31]. It has been suggested that the elevated succinic acid may act as a marker of brain injury under HI [32]. Also, succinic acid is an important vascular growth factor and might contribute to the repair of brain injury [33]. As an alternate energy source, the increased branched-chain amino acid like valine and isoleucine, and carnitine have been found in our study, which may meet the emergency needs of the system in case of energy reduction [26]. Also, the reduced creatinine levels found in the blood may be associated with impaired energy transfer systems [27].

Some metabolites like amino acid usually have a dual role acting as a source of energy metabolism and as precursors for neurotransmitter synthesis. Similar to the results of UCB metabolomics, metabolic pathways associated with the synthesis of serotonin and dopamine such as tryptophan metabolism, tyrosine metabolism, and phenylalanine metabolism were altered in our research [34], suggesting the perturbation of brain neurotransmitter synthesis [35, 36]. Increased inhibitory neurotransmitters like taurine and alanine and elevated excitatory neurotransmitters like glutamic acid have been identified in higher abundance in the HIE group. This excitotoxicity may result from the depolarization of the cell membrane and the release of a large amount of glutamate under free radical damage [3] and further cause more cell damage [9, 37]. The results of the diagnostic analysis showed that 14 candidate metabolites that were disordered in amino acid metabolism and energy metabolism had high specificity and sensitivity in the diagnosis of HIE. In particular, alpha-ketoglutaric acid had a good ability to distinguish the severity of HIE. It may be a good and broad-spectrum candidate biomarker since it was involved in 181 changed pathways.

### 4.2. Other Metabolism

Pyrimidine metabolism and ketone body metabolism were aberrant in blood plasma metabolomics, which were also present in the UCB metabolomics [34]. The decrease of the arachidonic acid level discovered in the plasma may increase the free radical damage and reduce neuronal repair capacity in HIE because this metabolite is an inflammatory mediator and vasodilator which can defend against oxidative stress in the brain and participates in the growth and repair of neurons [38, 39]. As a lipid metabolism-related molecule, the elevated myo-inositol in PB plasma may indicate the impairment in lipid metabolism in HIE, which was also present at urinary metabolomics [31]. Besides, metabolite set enrichment analysis also revealed several disturbed pathways closely related to brain dysfunction [40, 41], including GABA synthesis, release, reuptake and degradation, fatty acid biosynthesis, and regulation of development of the central nervous system [42]. This further suggested that each metabolic pathway does not function independently but interacts with each other to perform its biological function, so the regulatory mechanism of this complex metabolic network needs to be further investigated.

### 4.3. Heterogeneity Analysis on the Metabolic Profile of HIE

In terms of different species, compared with the finding in mouse [27, 28], piglet [30, 43–45], and human primate [29], we found that only several coexpressed metabolites were present in the PB plasma of HIE. For the different biosample types, there were also few coexpressed metabolites among human PB, UCB, and urine [26, 31, 34, 46]. To sum up, this may result from (1) the intrinsic heterogeneity of biosamples determined by species, sample type, sampling time, and inclusion and exclusion criteria; (2) the difference in specificity and sensitivity of the different analytical platform including GC/MS, LC/MS, H-NMR, and self-established platform; and (3) the different means of data processing. Normalization is a challenging and essential part of data analysis. Different methods of standardization directly affected the results of data analysis [25]. In our study, the standard normalization method and EigenMS method were used to normalize the data. For the standard normalization method, the results showed that the PCA could not separate both groups, and only 11 significantly differential metabolites were matched from the KEGG database. In contrast, the EigenMS method is more suitable for processing data in our research, and 52 differential metabolites were identified. 6 significantly coexpressed differential metabolites were identified between both methods (Supplementary Figure 3). This suggested that the formulation of guidelines for standardized processing of mass spectrometry data is also an urgent problem to be solved in the future.

### 4.4. Limitation

The study has its limitations. First, the sample size is relatively small, and the result of diagnostic analysis did not rule out the interference of confounding factors including the weight, gender, mode of delivery, and gestational age. So, multicenter large-scale validation is required to verify the results. Second, factors of preanalysis such as the fasting before sampling were not well-controlled because of the urgency and uncontrollability of time during the delivery process. Third, the long-term follow-up with scientific outcome assessment will be necessary in future clinical research. In our study, we only conducted a telephone follow-up every 6 months for the included cases due to the economic limitation and the imperfect medical system. The outcome data were summarized as follows: 3 cases died, 1 case refused to return visit, 1 case failed to connect, and the remaining 19 cases had no symptoms of neurodevelopmental retardation. The third stage during the progression of HIE may last for months and even years. Finally, the integrated analysis of multiomics will be essential to explore useful biomarkers and new therapeutic targets for HIE diagnosis and treatment.

## 5. Conclusions

Generally, HIE is a complex disease accompanied by multiple organ injury. The blood plasma metabolic profile could comprehensively reflect the changes of the whole body under HI injury. Most of the altered pathways were associated with amino acid metabolism, energy metabolism, biosynthesis of neurotransmitters, tryptophan, and pyrimidine metabolism. 14 candidate metabolites showed good diagnostic potential. But, the verification of large and independent cohorts is necessary. Meanwhile, the clinical application and standardization of mass spectrometry is a great challenge and dilemma at present.

## Figures and Tables

**Table 1:** Baseline characteristics of the participants (*n* = 48).

	Control (*n* = 24)	HIE (*n* = 24)	*p* value
Gestational age (weeks, mean)	40 ± 1	38 ± 3	0.004
Gender (male, *n*, %)	13 (54.2%)	16 (66.7)	0.376
Birth weight (g)	3401 ± 283	2647 ± 731	<0.001
Method of delivery			0.080
Vaginal	9 (37.5)	15 (62.5)	
C-section	15 (62.5)	9 (37.5)	
Apgar 1 (mean (min–max))	9 (9-10)	5 (2-8)	<0.001
Apgar 5 (mean (min–max))	10 (9-10)	7 (5-8)	<0.001
Maternal ethnicity			
Han	24	24	1.00
Maternal age (years)	29 ± 4	31 ± 6	0.180
Inflammatory markers			
Leucocyte count (10^9^ cells/L)	11.92 ± 6.07	15.57 ± 6.30	0.047
Neutrophil count (10^9^ cells/L)	7.89 ± 5.41	8.83 ± 5.47	0.551
Lymphocyte count (10^9^ cells/L)	2.81 ± 0.89	5.55 ± 3.27	0.001
Monocyte count (10^9^ cells/L)	0.92 ± 0.45	0.96 ± 0.44	0.795
Other index			
RBC (10^12^ cells/L)	4.85 ± 0.47	4.87 ± 0.54	0.892
Hb (g/L)	170.04 ± 16.73	172.50 ± 18.79	0.634
HCT (%)	49.17 ± 8.92	51.88 ± 8.17	0.278
PLT (10^9^/L)	274 ± 72	279 ± 106	0.869
MCV (fL)	106.34 ± 4.14	108.96 ± 7.38	0.138
MCH (pg)	35.08 ± 1.30	35.56 ± 2.52	0.410
MCHC (g/L)	330.04 ± 10.35	326.54 ± 17.60	0.407
MPV (fL)	7.35 ± 1.44	8.02 ± 0.67	0.047
CV (%)	14.75 ± 0.85	14.78 ± 1.75	0.942
SD (fL)	59.96 ± 4.16	61.94 ± 9.61	0.361
PDW	16.53 ± 0.89	16.10 ± 0.42	0.041
PCT	0.21 ± 0.05	0.22 ± 0.07	0.637

Data presented as mean ± s.d. or percentage or median. HIE: hypoxic-ischaemic encephalopathy.

## Data Availability

The data is available from the corresponding author on reasonable request.

## Supplementary Materials

Supplementary Materials Supplementary Table 1: entropy results of each group of samples with seven normalization methods. Supplementary Table 2: key differential metabolites detected by gas chromatography-mass spectrometry (GC-MS) for HIE vs. CON. Supplementary Table 3: the metabolic pathways based on seven databases. Supplementary Table 4: the overlapped pathways based on seven databases. Supplementary Figure 1: the quality control diagram. Supplementary Figure 2: the prediction of diagnostic value of differential candidate metabolites on the severity of HIE. Supplementary Figure 3: comparison of differential metabolites screened by different data standardization methods.
