# Supplementary material for: Blood Plasma Metabolic Profile of Newborns with Hypoxic-Ischaemic Encephalopathy by GC-MS
Source: Biomed Res Int. 2021 Jun 24;2021:6677271. doi: 10.1155/2021/6677271 (PMC8249136; doi:10.1155/2021/6677271)
Supplement: Supplementary Materials — Supplementary Table 1: entropy results of each group of samples with seven normalization methods. Supplementary Table 2: key differential metabolites detected by gas chromatography-mass spectrometry (GC-MS) for HIE vs. CON. Supplementary Table 3: the metabolic pathways based on seven databases. Supplementary Table 4: the overlapped pathways based on seven databases. Supplementary Figure 1: the quality control diagram. Supplementary Figure 2: the prediction of diagnostic value of differential candidate metabolites on the severity of HIE. Supplementary Figure 3: comparison of differential metabolites screened by different data standardization methods. [file 6677271.f1.doc]

**Blood plasma metabolic profile of newborns with hypoxic-ischaemic encephalopathy by GC-MS**

Running title: Screening non-invasive biomarkers for early diagnosis of HIE

Yanjuan Jia^1,2#^, Xiaoni, Jia^3#^, Hui Xu^1,2^, Lan Gao^4^, Chaojun Wei^1,2^, Yonghong Li^1,2^, Xia Liu^3^, Xiaoling Gao^1,2*^, Li Wei^5*^

1NHC Key Laboratory of Diagnosis and Therapy of Gastrointestinal Tumor, Gansu Provincial Hospital, Lanzhou, China, 730000

2The Institute of Clinical Research and Translational Medicine, Gansu Provincial Hospital, Lanzhou, China, 730000

3 The Neonatal Department, Qingyang People's Hospital, Qingyang, China, 73000

4 The Department of life science, Lanzhou University, Lanzhou, China, 730000

5 The Laboratory Center, Gansu Provincial Hospital, Lanzhou, China, 730000

# Yanjuan Jia and Xiaoni Jia are listed as co-first authors

Corresponding author:

*Xiaoling Gao and Li Wei are listed as co-corresponding authors

Dr.Xiaoling Gao, Ph.D., 204#, Donggang West Road, Chengguan District, Lanzhou, Gansu, China, 730000. E-mail: gaoxl008@hotmail.com; Phone: +86-931-8281222

Dr.Li Wei, 204#, Donggang West Road, Chengguan District, Lanzhou, Gansu, China, 730000. E-mail: peixiaoqiao@126.com; Phone: +86-931-8281883

**Supplementary Table 1 Entropy Results of Each Group of Samples with Seven Normalization Methods**

|  | **Entropies** | | |
| --- | --- | --- | --- |
| **Methods** | **HIE Group** | **Control Group** | **QC** |
| **None** | 2.94 | 3.16 | 2.08 |
| **Median** | 3.00 | 2.92 | 2.08 |
| **Standard** | 3.06 | 2.82 | 2.08 |
| **VSN** | 3.06 | 2.97 | 2.08 |
| **RUV-random** | 3.13 | 3.08 | 2.08 |
| **QC-SVR** | 3.08 | 2.96 | 2.08 |
| **EigenMS** | 3.18 | 3.16 | 2.08 |
| **QC-RLSC** | 3.17 | 3.10 | 2.07 |

VSN: Variance stabilizing normalization; QC-SVR: quality control sample-based support vector regression; RUN-random: Remove Unwanted Variation-Random; QC-RLSC: quality control-based robust LOESS signal correction.

**Supplementary Table 2 Key differential metabolites detected by gas chromatography-mass spectrometry (GC-MS) for HIE *vs* CON**

| **Metabolite** | **Subclass** | **RT (minute)** | **Mass** | **KEGG** | **VIP** | **Adjusted**  **P value** | **Log FC** | **Change Direction** |
| --- | --- | --- | --- | --- | --- | --- | --- | --- |
| Phthalic acid | Benzoic acids and derivatives | 5.35 | 174 | C01606 | 1.87 | 2.25E-10 | 0.25 | Up |
| Succinic acid | Dicarboxylic acids and derivatives | 8.96 | 198 | C00042 | 1.94 | 2.20E-10 | 0.15 | Up |
| Taurine | Organosulfonic acids and derivatives | 5.75 | 116 | C00245 | 1.90 | 1.17E-10 | 0.14 | Up |
| Pyruvic acid | Alpha-keto acids and derivatives | 7.27 | 247 | C00022 | 2.15 | 1.00E-16 | 0.12 | Up |
| Cystine | Amino acids, peptides, and analogues | 10.12 | 156 | C00491 | 1.03 | 2.55E-03 | 0.11 | Up |
| Cellobiose 2 | Carbohydrates and carbohydrate conjugates | 10.04 | 174 | C06422 | 1.08 | 6.58E-04 | 0.11 | Up |
| Hydroxylamine | Not Available | 9.33 | 218 | C00192 | 1.57 | 1.36E-06 | 0.10 | Up |
| Isomaltose 1 | Carbohydrates and carbohydrate conjugates | 11.01 | 218 | C00252 | 1.25 | 1.78E-04 | 0.10 | Up |
| L-Malic acid | Beta hydroxy acids and derivatives | 14.01 | 204 | C00149 | 2.23 | 1.43E-19 | 0.09 | Up |
| D-(glycerol 1-phosphate) | Glycerophosphates | 10.02 | 299 | C00093 | 1.19 | 3.54E-04 | 0.09 | Up |
| Glutamic acid | Dicarboxylic acids and derivatives | 8.86 | 218 | C00025 | 1.96 | 1.12E-11 | 0.09 | Up |
| Tartronic acid | Carbohydrates and carbohydrate conjugates | 8.06 | 86 | [C02287](https://www.kegg.jp/dbget-bin/www_bget?cpd:C02287) | 1.49 | 3.62E-06 | 0.08 | Up |
| Alanine 1 | Amino acids, peptides, and analogues | 11.74 | 217 | [C00041](https://www.kegg.jp/dbget-bin/www_bget?cpd:C00041) | 2.08 | 6.30E-14 | 0.08 | Up |
| 2,6-Diaminopimelic acid 2 | Amino acids, peptides, and analogues | 7.19 | 142 | C00666 | 1.84 | 2.93E-09 | 0.08 | Up |
| Threonic acid | Carbohydrates and carbohydrate conjugates | 8.41 | 73 | C01620 | 1.63 | 3.10E-06 | 0.08 | Up |
| beta-Alanine 2 | Amino acids, peptides, and analogues | 6.76 | 117 | C00099 | 1.45 | 6.24E-06 | 0.07 | Up |
| alpha-Ketoglutaric acid | Medium-chain keto acids and derivatives | 7.13 | 158 | [C00026](https://www.kegg.jp/dbget-bin/www_bget?cpd:C00026) | 1.44 | 6.70E-06 | 0.06 | Up |
| Ornithine 2 | Amino acids, peptides, and analogues | 7.35 | 189 | C00077 | 1.45 | 1.42E-05 | 0.05 | Up |
| Indolelactate 2 | Indolyl carboxylic acids and derivatives | 6.63 | 156 | [C02043](https://www.kegg.jp/dbget-bin/www_bget?cpd:C02043) | 1.65 | 2.05E-07 | 0.05 | Up |
| L-Cysteine | Amino acids, peptides, and analogues | 7.13 | 158 | C00097 | 1.69 | 9.38E-08 | 0.05 | Up |
| Norvaline | Amino acids, peptides, and analogues | 10.58 | 174 | C01799 | 1.13 | 6.46E-04 | 0.04 | Up |
| Phenylalanine 1 | Amino acids, peptides, and analogues | 12.35 | 117 | C00079 | 1.70 | 8.69E-08 | 0.04 | Up |
| Valine | Amino acids, peptides, and analogues | 10.44 | 217 | C00183 | 1.34 | 4.93E-05 | 0.03 | Up |
| Malonic acid 1 | Dicarboxylic acids and derivatives | 7.42 | 156 | C00383 | 1.56 | 2.51E-06 | 0.03 | Up |
| D-Glyceric acid | Carbohydrates and carbohydrate conjugates | 11.17 | 202 | C00258 | 1.32 | 9.33E-05 | 0.03 | Up |
| Dihydroxyacetone | Carbohydrates and carbohydrate conjugates | 13.48 | 364 | C00184 | 1.48 | 6.89E-06 | 0.03 | Up |
| Myo-inositol | Alcohols and polyols | 6.16 | 147 | C00137 | 1.33 | 1.14E-04 | 0.03 | Up |
| Carnitine | Hybrid peptides | 10.67 | 307 | C00318 | 1.37 | 6.83E-05 | 0.03 | Up |
| Pyrrole-2-carboxylic acid | Pyrrole carboxylic acids and derivatives | 10.61 | 103 | C05942 | 1.18 | 6.08E-04 | 0.03 | Up |
| Isoleucine | Amino acids, peptides, and analogues | 12.32 | 337 | C00407 | 1.02 | 2.72E-03 | 0.02 | Up |
| Proline | Quaternary ammonium salts | 7.61 | 117 | C00148 | 1.33 | 8.95E-05 | 0.02 | Up |
| Octanal 1 | Carbonyl compounds | 8.84 | 292 | C01545 | 1.04 | 1.04E-03 | 0.01 | Up |
| 2-Deoxyuridine | Pyrimidine 2'-deoxyribonucleosides | 6.78 | 144 | C00526 | 1.14 | 3.74E-04 | 0.01 | Up |
| Asparagine 4 | Steroidal glycosides | 6.52 | 57 | C01905 | 1.22 | 2.69E-04 | -0.03 | Down |
| Arachidic acid | Fatty acids and conjugates | 13.74 | 127 | C06425 | 1.05 | 3.15E-03 | -0.03 | Down |
| Aminomalonic acid | Amino acids, peptides, and analogues | 10.55 | 132 | C00872 | 1.22 | 8.86E-05 | -0.03 | Down |
| Myristic acid | Fatty acids and conjugates | 13.30 | 187 | C06424 | 1.40 | 5.39E-05 | -0.04 | Down |
| N-Acetyl-5-hydroxytryptamine 2 | Benzoic acids and derivatives | 5.73 | 154 | [C00978](https://www.kegg.jp/dbget-bin/www_bget?cpd:C00978) | 1.47 | 1.54E-05 | -0.04 | Down |
| Tyrosine 1 | Amino acids, peptides, and analogues | 11.45 | 117 | [C00082](https://www.kegg.jp/dbget-bin/www_bget?cpd:C00082) | 1.36 | 5.64E-05 | -0.04 | Down |
| Arbutin | Carbohydrates and carbohydrate conjugates | 7.63 | 194 | C06186 | 1.08 | 1.95E-03 | -0.04 | Down |
| Creatine | Amino acids, peptides, and analogues | 8.56 | 115 | C00300 | 1.46 | 3.46E-06 | -0.05 | Down |
| Elaidic acid | Fatty acids and conjugates | 6.54 | 89 | C00712 | 1.50 | 3.64E-06 | -0.08 | Down |
| 3-Hydroxybutyric acid | Beta hydroxy acids and derivatives | 10.40 | 160 | C01089 | 1.34 | 5.15E-05 | -0.08 | Down |
| Pelargonic acid | Fatty acids and conjugates | 8.61 | 146 | C01601 | 1.90 | 2.01E-09 | -0.09 | Down |
| Linoleic acid | Lineolic acids and derivatives | 8.12 | 327 | C01595 | 1.22 | 2.52E-04 | -0.09 | Down |
| Geraniol | Monoterpenoids | 8.21 | 73 | C09871 | 1.85 | 1.04E-09 | -0.11 | Down |
| Glutamine 1 | Amino acids, peptides, and analogues | 7.30 | 240 | C00064 | 1.96 | 2.67E-11 | -0.12 | Down |
| Palmitoleic acid | Fatty acids and conjugates | 9.52 | 70 | C08362 | 1.69 | 9.38E-08 | -0.13 | Down |
| Lactose 2 | Carbohydrates and carbohydrate conjugates | 9.70 | 282 | [C00243](https://www.kegg.jp/dbget-bin/www_bget?cpd:C00243) | 1.60 | 4.36E-07 | -0.13 | Down |
| Indole-3-acetic acid | Indolyl carboxylic acids and derivatives | 11.91 | 174 | C00954 | 1.81 | 1.90E-09 | -0.14 | Down |
| Tagatose 1 | Carbohydrates and carbohydrate conjugates | 10.07 | 217 | C00795 | 1.82 | 8.35E-10 | -0.15 | Down |
| Sucrose | Carbohydrates and carbohydrate conjugates | 11.77 | 227 | C00089 | 1.74 | 2.42E-09 | -0.19 | Down |

KEGG, Kyoto Encyclopedia of Genes and Genomes; RT, retention time; FC, Fold change; HIE, Hypoxic-ischaemic encephalopathy.

**Supplementary Table 3 The metabolic pathways based on seven databases**

| **PathwayName** | **PathwaySource** | **Adjusted p value** |
| --- | --- | --- |
| &beta;-alanine degradation | HumanCyc | 0.001029337 |
| (S)-reticuline biosynthesis | HumanCyc | 0.002561249 |
| [2Fe-2S] iron-sulfur cluster biosynthesis | HumanCyc | 0.005973146 |
| <i>S</i>-methyl-5-thio-&alpha;-D-ribose 1-phosphate degradation | HumanCyc | 0.03107328 |
| 2-ketoglutarate dehydrogenase complex deficiency | SMPDB | 0.001789699 |
| 2-Methyl-3-Hydroxybutryl CoA Dehydrogenase Deficiency | SMPDB | 0.001678113 |
| 3-Hydroxy-3-Methylglutaryl-CoA Lyase Deficiency | SMPDB | 0.001678113 |
| 3-hydroxyisobutyric acid dehydrogenase deficiency | SMPDB | 0.001678113 |
| 3-hydroxyisobutyric aciduria | SMPDB | 0.001678113 |
| 3-Methylcrotonyl Coa Carboxylase Deficiency Type I | SMPDB | 0.001678113 |
| 3-Methylglutaconic Aciduria Type I | SMPDB | 0.001678113 |
| 3-Methylglutaconic Aciduria Type III | SMPDB | 0.001678113 |
| 3-Methylglutaconic Aciduria Type IV | SMPDB | 0.001678113 |
| 4-aminobutyrate degradation | HumanCyc | 0.000568964 |
| 4-hydroxybenzoate biosynthesis | HumanCyc | 0.002885199 |
| 4-hydroxyproline degradation | HumanCyc | 0.002885199 |
| 5-aminoimidazole ribonucleotide biosynthesis | HumanCyc | 0.045573358 |
| 5-oxoprolinase deficiency | SMPDB | 0.005710518 |
| 5-Oxoprolinuria | SMPDB | 0.005710518 |
| Activated PKN1 stimulates transcription of AR (androgen receptor) regulated genes KLK2 and KLK3 | Wikipathways | 0.016442087 |
| Activated PKN1 stimulates transcription of AR (androgen receptor) regulated genes KLK2 and KLK3 | Reactome | 0.016442087 |
| acyl-CoA hydrolysis | HumanCyc | 0.021888955 |
| Alanine and aspartate metabolism | Wikipathways | 0.003408483 |
| Alanine Metabolism | SMPDB | 0.000224347 |
| Alcoholism | KEGG | 0.014312993 |
| ALKBH2 mediated reversal of alkylation damage | Reactome | 0.007930449 |
| ALKBH3 mediated reversal of alkylation damage | Reactome | 0.007930449 |
| Amikacin Action Pathway | SMPDB | 0.000338944 |
| Amine-derived hormones | Wikipathways | 0.020471969 |
| Amine-derived hormones | Reactome | 0.020471969 |
| Amino acid conjugation | Wikipathways | 0.004340967 |
| Amino Sugar Metabolism | SMPDB | 0.01426441 |
| Aminosugars metabolism | EHMN | 0.00419552 |
| Ammonia Recycling | SMPDB | 0.001249547 |
| Amphetamine addiction | KEGG | 0.014312993 |
| Arbekacin Action Pathway | SMPDB | 0.000338944 |
| Arginine and Proline Metabolism | SMPDB | 0.000149892 |
| Arginine and proline metabolism | KEGG | 0.00080559 |
| Arginine biosynthesis | KEGG | 0.000552417 |
| Arginine: Glycine Amidinotransferase Deficiency (AGAT Deficiency) | SMPDB | 0.000149892 |
| Ascorbate and aldarate metabolism | KEGG | 0.005608826 |
| asparagine biosynthesis | HumanCyc | 0.001029337 |
| asparagine degradation | HumanCyc | 0.014312993 |
| aspartate biosynthesis | HumanCyc | 0.002741214 |
| Aspartate Metabolism | SMPDB | 0.001938973 |
| Astrocytic Glutamate-Glutamine Uptake And Metabolism | Reactome | 0.005973146 |
| Azithromycin Action Pathway | SMPDB | 0.000338944 |
| Beta-Alanine Metabolism | SMPDB | 0.016011987 |
| Beta-Ketothiolase Deficiency | SMPDB | 0.001678113 |
| Beta-mercaptolactate-cysteine disulfiduria | SMPDB | 0.000847392 |
| Bile salt and organic anion SLC transporters | Reactome | 0.034368711 |
| Biogenic Amine Synthesis | Wikipathways | 0.000224347 |
| Biosynthesis of unsaturated fatty acids | KEGG | 0.047013625 |
| Branched-chain amino acid catabolism | Wikipathways | 0.00067979 |
| Branched-chain amino acid catabolism | Reactome | 0.000745623 |
| Butanoate metabolism | EHMN | 0.000295719 |
| Butanoate metabolism | KEGG | 0.000411757 |
| Canavan Disease | SMPDB | 0.001938973 |
| Carnitine Synthesis | SMPDB | 0.004508299 |
| Carnitine synthesis | Reactome | 0.028158902 |
| Carnosinuria, carnosinemia | SMPDB | 0.016011987 |
| Cellular response to hypoxia | Reactome | 0.002741214 |
| Cellular Senescence | Reactome | 0.016442087 |
| Chloramphenicol Action Pathway | SMPDB | 0.000338944 |
| Chromatin modifying enzymes | Wikipathways | 0.03107328 |
| Chromatin modifying enzymes | Reactome | 0.03107328 |
| Chromatin organization | Reactome | 0.03107328 |
| Citrate cycle | INOH | 0.002143532 |
| Citrate cycle (TCA cycle) | KEGG | 0.000338944 |
| Citric Acid Cycle | SMPDB | 0.001789699 |
| Citric acid cycle (TCA cycle) | Reactome | 0.010522188 |
| Clarithromycin Action Pathway | SMPDB | 0.000338944 |
| Class A/1 (Rhodopsin-like receptors) | Reactome | 0.001029337 |
| Class C/3 (Metabotropic glutamate/pheromone receptors) | Reactome | 0.008538669 |
| Clindamycin Action Pathway | SMPDB | 0.000338944 |
| Clomocycline Action Pathway | SMPDB | 0.000338944 |
| Cocaine addiction | KEGG | 0.010183594 |
| Collagen biosynthesis and modifying enzymes | Wikipathways | 0.012539548 |
| Collagen biosynthesis and modifying enzymes | Reactome | 0.012539548 |
| Collagen formation | Reactome | 0.021888955 |
| Condensation of Prophase Chromosomes | Reactome | 0.014312993 |
| Congenital lactic acidosis | SMPDB | 0.001789699 |
| creatine biosynthesis | HumanCyc | 0.010183594 |
| Creatine deficiency, guanidinoacetate methyltransferase deficiency | SMPDB | 0.000149892 |
| Creatine metabolism | Reactome | 0.028158902 |
| Cysteine and methionine metabolism | KEGG | 0.013236961 |
| Cysteine Metabolism | SMPDB | 0.000847392 |
| Cystinosis, ocular nonnephropathic | SMPDB | 0.000847392 |
| Degradation of cysteine and homocysteine | Reactome | 0.013440308 |
| Degradation of GABA | Reactome | 0.000568964 |
| Demeclocycline Action Pathway | SMPDB | 0.000338944 |
| D-Glutamine and D-glutamate metabolism | KEGG | 0.001304747 |
| Digestion of dietary carbohydrate | Reactome | 0.002143532 |
| DNA Damage Reversal | Wikipathways | 0.010183594 |
| DNA Damage Reversal | Reactome | 0.014312993 |
| Doxycycline Action Pathway | SMPDB | 0.000338944 |
| Erythromycin Action Pathway | SMPDB | 0.000338944 |
| fatty acid &alpha;-oxidation | HumanCyc | 0.045573358 |
| fatty acid activation | HumanCyc | 0.0003741 |
| Fatty Acid Biosynthesis | SMPDB | 0.01426441 |
| Fatty acid biosynthesis | KEGG | 0.039212358 |
| Fatty acid, triacylglycerol, and ketone body metabolism | Reactome | 0.028329555 |
| Free fatty acid receptors | Reactome | 0.007028988 |
| Fumarase deficiency | SMPDB | 0.001789699 |
| G alpha (i) signalling events | Reactome | 0.006314506 |
| G alpha (q) signalling events | Reactome | 0.033838654 |
| G(M2)-Gangliosidosis: Variant B, Tay-sachs disease | SMPDB | 0.01426441 |
| GABA shunt | HumanCyc | 0.001647243 |
| GABA synthesis, release, reuptake and degradation | Wikipathways | 0.000184552 |
| GABA synthesis, release, reuptake and degradation | Reactome | 0.000184552 |
| GABA-Transaminase Deficiency | SMPDB | 0.016011987 |
| Galactose metabolism | KEGG | 0.00483264 |
| Galactose Metabolism | SMPDB | 0.017930539 |
| Galactosemia | SMPDB | 0.017930539 |
| Gamma-Glutamyltransferase Deficiency | SMPDB | 0.005710518 |
| Gamma-glutamyl-transpeptidase deficiency | SMPDB | 0.005710518 |
| Gentamicin Action Pathway | SMPDB | 0.000338944 |
| Glucagon signaling pathway | KEGG | 0.000847392 |
| Gluconeogenesis | Reactome | 0.001938973 |
| Glucose Alanine cycle | Wikipathways | 0.004340967 |
| Glucose metabolism | Reactome | 0.005608826 |
| glutamate biosynthesis/degradation | HumanCyc | 0.012539548 |
| Glutamate Neurotransmitter Release Cycle | Reactome | 0.010183594 |
| Glutamatergic synapse | KEGG | 0.010183594 |
| glutamine biosynthesis | HumanCyc | 0.007930449 |
| glutamine degradation/glutamate biosynthesis | HumanCyc | 0.002741214 |
| glutathione biosynthesis | HumanCyc | 0.012539548 |
| Glutathione Metabolism | SMPDB | 0.005710518 |
| Glutathione metabolism | KEGG | 0.020471969 |
| Glutathione metabolism | Wikipathways | 0.042003707 |
| Glutathione synthesis and recycling | Reactome | 0.03107328 |
| Glutathione Synthetase Deficiency | SMPDB | 0.005710518 |
| glutathione-mediated detoxification | HumanCyc | 0.024961118 |
| Glycerolipid metabolism | KEGG | 0.016746165 |
| Glycerophospholipid catabolism | Reactome | 0.016442087 |
| Glycerophospholipid metabolism | EHMN | 0.002171338 |
| glycine biosynthesis | HumanCyc | 0.002741214 |
| Glycine, serine and threonine metabolism | KEGG | 0.006620922 |
| GPCR downstream signaling | Reactome | 0.00169583 |
| GPCR downstream signaling | Wikipathways | 0.004712619 |
| Guanidinoacetate Methyltransferase Deficiency (GAMT Deficiency) | SMPDB | 0.000149892 |
| guanosine nucleotides <i>de novo</i> biosynthesis | HumanCyc | 0.034368711 |
| guanosine ribonucleotides <i>de novo</i> biosynthesis | HumanCyc | 0.028158902 |
| HDMs demethylate histones | Reactome | 0.004340967 |
| HIF-1 signaling pathway | KEGG | 0.028158902 |
| HIF1A and PPARG regulation of glycolysis | Wikipathways | 0.014312993 |
| Histidine metabolism | EHMN | 0.03099117 |
| Hyperornithinemia with gyrate atrophy (HOGA) | SMPDB | 0.000149892 |
| Hyperornithinemia-hyperammonemia-homocitrullinuria [HHH-syndrome] | SMPDB | 0.000149892 |
| Hyperprolinemia Type I | SMPDB | 0.000149892 |
| Hyperprolinemia Type II | SMPDB | 0.000149892 |
| Hypoacetylaspartia | SMPDB | 0.001938973 |
| Incretin synthesis, secretion, and inactivation | Wikipathways | 0.005710518 |
| Isobutyryl-coa dehydrogenase deficiency | SMPDB | 0.001678113 |
| isoleucine degradation | HumanCyc | 0.002561249 |
| Isovaleric acidemia | SMPDB | 0.001678113 |
| Isovaleric Aciduria | SMPDB | 0.001678113 |
| Josamycin Action Pathway | SMPDB | 0.000338944 |
| Kanamycin Action Pathway | SMPDB | 0.000338944 |
| ketolysis | HumanCyc | 0.014312993 |
| Ketone Body Metabolism | SMPDB | 0.021888955 |
| Ketone body metabolism | Reactome | 0.03107328 |
| Lactic Acidemia | SMPDB | 0.000224347 |
| L-arginine:glycine amidinotransferase deficiency | SMPDB | 0.000149892 |
| L-carnitine biosynthesis | HumanCyc | 0.001808517 |
| L-dopa degradation | HumanCyc | 0.019163276 |
| leucine degradation | HumanCyc | 0.045573358 |
| L-glutamine tRNA biosynthesis | HumanCyc | 0.012539548 |
| Lincomycin Action Pathway | SMPDB | 0.000338944 |
| L-kynurenine degradation | HumanCyc | 0.012539548 |
| Lymecycline Action Pathway | SMPDB | 0.000338944 |
| Lysine degradation | INOH | 0.017930539 |
| lysine degradation I (saccharopine pathway) | HumanCyc | 0.03107328 |
| lysine degradation II (pipecolate pathway) | HumanCyc | 0.049671187 |
| Lysine metabolism | EHMN | 0.045387069 |
| M Phase | Reactome | 0.03811855 |
| malate-aspartate shuttle | HumanCyc | 0.00040716 |
| Malate-Aspartate Shuttle | SMPDB | 0.014312993 |
| Malonic Aciduria | SMPDB | 0.002741214 |
| Malonyl-coa decarboxylase deficiency | SMPDB | 0.002741214 |
| Maple Syrup Urine Disease | SMPDB | 0.001678113 |
| Metabolism | Reactome | 0.000100605 |
| Metabolism of carbohydrates | Reactome | 0.000708298 |
| Metabolism of nucleotides | Wikipathways | 0.012539548 |
| Metabolism of nucleotides | Reactome | 0.014476013 |
| Metabolism of vitamins and cofactors | Reactome | 0.00450245 |
| Metabolism of water-soluble vitamins and cofactors | Reactome | 0.023173555 |
| Metabolism of water-soluble vitamins and cofactors | Wikipathways | 0.042764493 |
| Methacycline Action Pathway | SMPDB | 0.000338944 |
| Methionine De Novo and Salvage Pathway | Wikipathways | 0.024755432 |
| Methylmalonate Semialdehyde Dehydrogenase Deficiency | SMPDB | 0.001678113 |
| Methylmalonic Aciduria | SMPDB | 0.001678113 |
| Methylmalonic Aciduria Due to Cobalamin-Related Disorders | SMPDB | 0.002741214 |
| Minocycline Action Pathway | SMPDB | 0.000338944 |
| Mitochondrial complex II deficiency | SMPDB | 0.001789699 |
| Mitochondrial Electron Transport Chain | SMPDB | 0.03811855 |
| Mitochondrial iron-sulfur cluster biogenesis | Wikipathways | 0.019163276 |
| Mitotic Prophase | Wikipathways | 0.03107328 |
| Mitotic Prophase | Reactome | 0.03107328 |
| molybdenum cofactor biosynthesis | HumanCyc | 0.021888955 |
| Molybdenum cofactor biosynthesis | Reactome | 0.045573358 |
| NAD <i>de novo</i> biosynthesis | HumanCyc | 0.007028988 |
| NAD biosynthesis from 2-amino-3-carboxymuconate semialdehyde | HumanCyc | 0.024961118 |
| NAD Biosynthesis II (from tryptophan) | Wikipathways | 0.006307734 |
| Neomycin Action Pathway | SMPDB | 0.000338944 |
| Netilmicin Action Pathway | SMPDB | 0.000338944 |
| Neurotransmitter Release Cycle | Wikipathways | 0.002568178 |
| Neurotransmitter uptake and Metabolism In Glial Cells | Wikipathways | 0.005973146 |
| Neurotransmitter uptake and Metabolism In Glial Cells | Reactome | 0.005973146 |
| Nicotinate metabolism | Reactome | 0.033838654 |
| Nitrogen metabolism | KEGG | 0.00396906 |
| One carbon donor | Wikipathways | 0.006307734 |
| Organic anion transporters | Reactome | 0.002143532 |
| ornithine <i>de novo </i> biosynthesis | HumanCyc | 0.002143532 |
| Ornithine Aminotransferase Deficiency (OAT Deficiency) | SMPDB | 0.000149892 |
| Oxidation of Branched Chain Fatty Acids | SMPDB | 0.007899987 |
| Oxidative Stress Induced Senescence | Wikipathways | 0.010183594 |
| Oxidative Stress Induced Senescence | Reactome | 0.010183594 |
| Oxygen-dependent asparagine hydroxylation of Hypoxia-inducible Factor Alpha | Reactome | 0.002741214 |
| Oxygen-dependent proline hydroxylation of Hypoxia-inducible Factor Alpha | Reactome | 0.002741214 |
| Oxytetracycline Action Pathway | SMPDB | 0.000338944 |
| Pantothenate and CoA biosynthesis | KEGG | 0.001091711 |
| Paromomycin Action Pathway | SMPDB | 0.000338944 |
| Phase II conjugation | Wikipathways | 0.000338944 |
| Phenylalanine and Tyrosine Metabolism | SMPDB | 0.001091711 |
| Phenylalanine degradation | INOH | 0.001808517 |
| phenylalanine degradation/tyrosine biosynthesis | HumanCyc | 0.016442087 |
| Phenylalanine metabolism | KEGG | 0.019284742 |
| Phenylketonuria | SMPDB | 0.001091711 |
| phospholipases | HumanCyc | 0.000345413 |
| Primary Hyperoxaluria Type I | SMPDB | 0.000224347 |
| Prolidase Deficiency (PD) | SMPDB | 0.000149892 |
| proline biosynthesis | HumanCyc | 0.024961118 |
| Proline catabolism | Reactome | 0.014312993 |
| proline degradation | HumanCyc | 0.014312993 |
| Prolinemia Type II | SMPDB | 0.000149892 |
| Propanoate Metabolism | SMPDB | 0.002741214 |
| Propionic Acidemia | SMPDB | 0.001678113 |
| Proton-coupled neutral amino acid transporters | Reactome | 0.002741214 |
| Proximal tubule bicarbonate reclamation | KEGG | 0.000224347 |
| Pyrimidine catabolism | Reactome | 0.002943849 |
| Pyrimidine metabolism | Reactome | 0.000309235 |
| Pyrimidine metabolism | EHMN | 0.004854411 |
| Pyrimidine metabolism | KEGG | 0.016442087 |
| Pyrimidine nucleotides nucleosides metabolism | INOH | 0.001568249 |
| Pyruvate Carboxylase Deficiency | SMPDB | 0.000224347 |
| Pyruvate dehydrogenase deficiency (E2) | SMPDB | 0.001789699 |
| Pyruvate dehydrogenase deficiency (E3) | SMPDB | 0.001789699 |
| Pyruvate metabolism | KEGG | 0.013440308 |
| Pyruvate metabolism and Citric Acid (TCA) cycle | Reactome | 0.003865944 |
| Regulation of Hypoxia-inducible Factor (HIF) by oxygen | Wikipathways | 0.002741214 |
| Regulation of Hypoxia-inducible Factor (HIF) by oxygen | Reactome | 0.002741214 |
| retinol biosynthesis | HumanCyc | 0.000338944 |
| Reversal of alkylation damage by DNA dioxygenases | Reactome | 0.012539548 |
| RHO GTPases activate PKNs | Reactome | 0.028158902 |
| Rolitetracycline Action Pathway | SMPDB | 0.000338944 |
| Roxithromycin Action Pathway | SMPDB | 0.000338944 |
| Salla Disease/Infantile Sialic Acid Storage Disease | SMPDB | 0.01426441 |
| serine and glycine biosynthesis | HumanCyc | 0.03811855 |
| Serine biosynthesis | Reactome | 0.021888955 |
| serine biosynthesis (phosphorylated route) | HumanCyc | 0.016442087 |
| Sialuria or French Type Sialuria | SMPDB | 0.01426441 |
| Sialuria or French Type Sialuria | SMPDB | 0.01426441 |
| Signal Transduction | Reactome | 0.000102875 |
| Spectinomycin Action Pathway | SMPDB | 0.000338944 |
| sphingomyelin metabolism/ceramide salvage | HumanCyc | 0.0003741 |
| sphingosine and sphingosine-1-phosphate metabolism | HumanCyc | 0.000496923 |
| Streptomycin Action Pathway | SMPDB | 0.000338944 |
| Succinyl CoA: 3-ketoacid CoA transferase deficiency | SMPDB | 0.021888955 |
| Sudden Infant Death Syndrome (SIDS) Susceptibility Pathways | Wikipathways | 0.024961118 |
| Sulfur amino acid metabolism | Wikipathways | 0.013845917 |
| Sulfur amino acid metabolism | Reactome | 0.014476013 |
| Sulfur metabolism | KEGG | 0.01426441 |
| Sulfur relay system | KEGG | 0.014312993 |
| superpathway of conversion of glucose to acetyl CoA and entry into the TCA cycle | HumanCyc | 0.002339063 |
| superpathway of methionine degradation | HumanCyc | 0.002339063 |
| superpathway of tryptophan utilization | HumanCyc | 0.000535761 |
| Synthesis of UDP-N-acetyl-glucosamine | Reactome | 0.049671187 |
| Synthesis of wybutosine at G37 of tRNA(Phe) | Wikipathways | 0.001808517 |
| Synthesis of wybutosine at G37 of tRNA(Phe) | Reactome | 0.002143532 |
| Taste transduction | KEGG | 0.014882788 |
| Taurine and Hypotaurine Metabolism | SMPDB | 0.019163276 |
| taurine biosynthesis | HumanCyc | 0.016442087 |
| Tay-Sachs Disease | SMPDB | 0.01426441 |
| TCA cycle | HumanCyc | 0.006307734 |
| TCA Cycle | Wikipathways | 0.007028988 |
| TCA cycle | EHMN | 0.017930539 |
| TCA Cycle and Deficiency of Pyruvate Dehydrogenase complex (PDHc) | Wikipathways | 0.000224347 |
| Telithromycin Action Pathway | SMPDB | 0.000338944 |
| TET1,2,3 and TDG demethylate DNA | Wikipathways | 0.012539548 |
| TET1,2,3 and TDG demethylate DNA | Reactome | 0.016442087 |
| Tetracycline Action Pathway | SMPDB | 0.000338944 |
| The citric acid (TCA) cycle and respiratory electron transport | Wikipathways | 0.003539463 |
| The citric acid (TCA) cycle and respiratory electron transport | Reactome | 0.008538669 |
| The oncogenic action of Fumarate | SMPDB | 0.000460292 |
| The oncogenic action of Succinate | SMPDB | 0.000411757 |
| the visual cycle I (vertebrates) | HumanCyc | 0.000460292 |
| Thiamine metabolism | KEGG | 0.013440308 |
| thio-molybdenum cofactor biosynthesis | HumanCyc | 0.005973146 |
| Thyroxine biosynthesis | Reactome | 0.034368711 |
| Tigecycline Action Pathway | SMPDB | 0.000338944 |
| Tobramycin Action Pathway | SMPDB | 0.000338944 |
| Transport of vitamins, nucleosides, and related molecules | Wikipathways | 0.012185304 |
| Transport of vitamins, nucleosides, and related molecules | Reactome | 0.01426441 |
| Trans-sulfuration pathway | Wikipathways | 0.000119151 |
| triacylglycerol degradation | HumanCyc | 0.000401069 |
| tRNA modification in the nucleus and cytosol | Reactome | 0.033838654 |
| tRNA processing | Reactome | 0.041253162 |
| Troleandomycin Action Pathway | SMPDB | 0.000338944 |
| Tryptophan Metabolism | SMPDB | 0.001858605 |
| Tryptophan metabolism | EHMN | 0.005125971 |
| tyrosine degradation | HumanCyc | 0.001647243 |
| Tyrosine metabolism | INOH | 0.000560855 |
| Tyrosine metabolism | EHMN | 0.000641544 |
| Tyrosinemia Type 2 (or Richner-Hanhart syndrome) | SMPDB | 0.001091711 |
| Tyrosinemia Type 3 (TYRO3) | SMPDB | 0.001091711 |
| UDP-<i>N</i>-acetyl-D-glucosamine biosynthesis II | HumanCyc | 0.016442087 |
| UMP biosynthesis | HumanCyc | 0.045573358 |
| Urea cycle and metabolism of amino groups | Wikipathways | 0.001414886 |
| Ureidopropionase deficiency | SMPDB | 0.016011987 |
| Utilization of Ketone Bodies | Reactome | 0.014312993 |
| UTP and CTP <i>de novo</i> biosynthesis | HumanCyc | 0.016442087 |
| UTP and CTP dephosphorylation I | HumanCyc | 0.028158902 |
| UTP and CTP dephosphorylation II | HumanCyc | 0.016442087 |
| valine degradation | HumanCyc | 0.004508299 |
| Valine Leucine Isoleucine degradation | INOH | 0.0003741 |
| Valine, leucine and isoleucine biosynthesis | KEGG | 0.006307734 |
| Valine, Leucine and Isoleucine Degradation | SMPDB | 0.001678113 |
| Valine, leucine and isoleucine degradation | EHMN | 0.013236961 |
| Vitamin B6 metabolism | INOH | 0.034368711 |
| Warburg Effect | SMPDB | 0.000258603 |

**Supplementary Table 4 The overlapped pathways based on seven databases**

| **PathwayName** | **PathwaySource** | **Adjusted p value** |
| --- | --- | --- |
| Amine-derived hormones | Wikipathways | 0.02184393 |
| Amine-derived hormones | Reactome | 0.02184393 |
| Arginine and proline metabolism | SMPDB | 0.000169435 |
| Arginine and proline metabolism | KEGG | 0.000909741 |
| Branched-chain amino acid catabolism | Wikipathways | 0.000749351 |
| Branched-chain amino acid catabolism | Reactome | 0.000826771 |
| Butanoate metabolism | EHMN | 0.000324822 |
| Butanoate metabolism | KEGG | 0.000453981 |
| Carnitine synthesis | SMPDB | 0.004844224 |
| Carnitine synthesis | Reactome | 0.029421309 |
| Chromatin modifying enzymes | Wikipathways | 0.032463715 |
| Chromatin modifying enzymes | Reactome | 0.032463715 |
| Collagen biosynthesis and modifying enzymes | Wikipathways | 0.013233928 |
| Collagen biosynthesis and modifying enzymes | Reactome | 0.013233928 |
| DNA damage reversal | Wikipathways | 0.010679381 |
| DNA damage reversal | Reactome | 0.015005167 |
| Fatty acid biosynthesis | SMPDB | 0.015005167 |
| Fatty acid biosynthesis | KEGG | 0.041448798 |
| GABA synthesis, release, reuptake and degradation | Wikipathways | 0.000198455 |
| GABA synthesis, release, reuptake and degradation | Reactome | 0.000198455 |
| Galactose metabolism | KEGG | 0.005288628 |
| Galactose metabolism | SMPDB | 0.019138727 |
| Glutathione metabolism | SMPDB | 0.006110026 |
| Glutathione metabolism | KEGG | 0.02184393 |
| Glutathione metabolism | Wikipathways | 0.0436532 |
| GPCR downstream signaling | Reactome | 0.001928725 |
| GPCR downstream signaling | Wikipathways | 0.005292849 |
| Ketone body metabolism | SMPDB | 0.022998703 |
| Ketone body metabolism | Reactome | 0.032463715 |
| Malate-aspartate shuttle | HumanCyc | 0.000433875 |
| Malate-aspartate shuttle | SMPDB | 0.015005167 |
| Metabolism of nucleotides | Wikipathways | 0.014031683 |
| Metabolism of nucleotides | Reactome | 0.016158452 |
| Metabolism of water-soluble vitamins and cofactors | Reactome | 0.025491653 |
| Metabolism of water-soluble vitamins and cofactors | Wikipathways | 0.045869986 |
| Molybdenum cofactor biosynthesis | HumanCyc | 0.022998703 |
| Molybdenum cofactor biosynthesis | Reactome | 0.047268162 |
| Neurotransmitter uptake and metabolism in glial Cells | Wikipathways | 0.006265896 |
| Neurotransmitter uptake and metabolism in glial Cells | Reactome | 0.006265896 |
| Oxidative stress induced senescence | Wikipathways | 0.010679381 |
| Oxidative stress induced senescence | Reactome | 0.010679381 |
| Pyrimidine metabolism | Reactome | 0.000346341 |
| Pyrimidine metabolism | EHMN | 0.005383761 |
| Pyrimidine metabolism | KEGG | 0.017530769 |
| Regulation of hypoxia-inducible factor (HIF) by oxygen | Wikipathways | 0.002924602 |
| Regulation of hypoxia-inducible factor (HIF) by oxygen | Reactome | 0.002924602 |
| Sialuria or french type sialuria | SMPDB | 0.015005167 |
| Sialuria or french type sialuria | SMPDB | 0.015005167 |
| Sulfur amino acid metabolism | Wikipathways | 0.015005167 |
| Sulfur amino acid metabolism | Reactome | 0.015726792 |
| Synthesis of wybutosine at G37 of tRNA(Phe) | Wikipathways | 0.001928725 |
| Synthesis of wybutosine at G37 of tRNA(Phe) | Reactome | 0.002306586 |
| TCA cycle | HumanCyc | 0.006747853 |
| TCA Cycle | Wikipathways | 0.00751813 |
| TCA cycle | EHMN | 0.019138727 |
| TET1,2,3 and TDG demethylate DNA | Wikipathways | 0.013233928 |
| TET1,2,3 and TDG demethylate DNA | Reactome | 0.017283383 |
| The citric acid (TCA) cycle and respiratory electron transport | Wikipathways | 0.003861324 |
| The citric acid (TCA) cycle and respiratory electron transport | Reactome | 0.009292887 |
| Transport of vitamins, nucleosides, and related molecules | Wikipathways | 0.013233928 |
| Transport of vitamins, nucleosides, and related molecules | Reactome | 0.015005167 |
| Tryptophan metabolism | SMPDB | 0.002058447 |
| Tryptophan metabolism | EHMN | 0.005683844 |
| Tyrosine metabolism | INOH | 0.000610806 |
| Tyrosine metabolism | EHMN | 0.000733552 |
| Valine, leucine and isoleucine degradation | SMPDB | 0.001858756 |
| Valine, leucine and isoleucine degradation | EHMN | 0.014358251 |


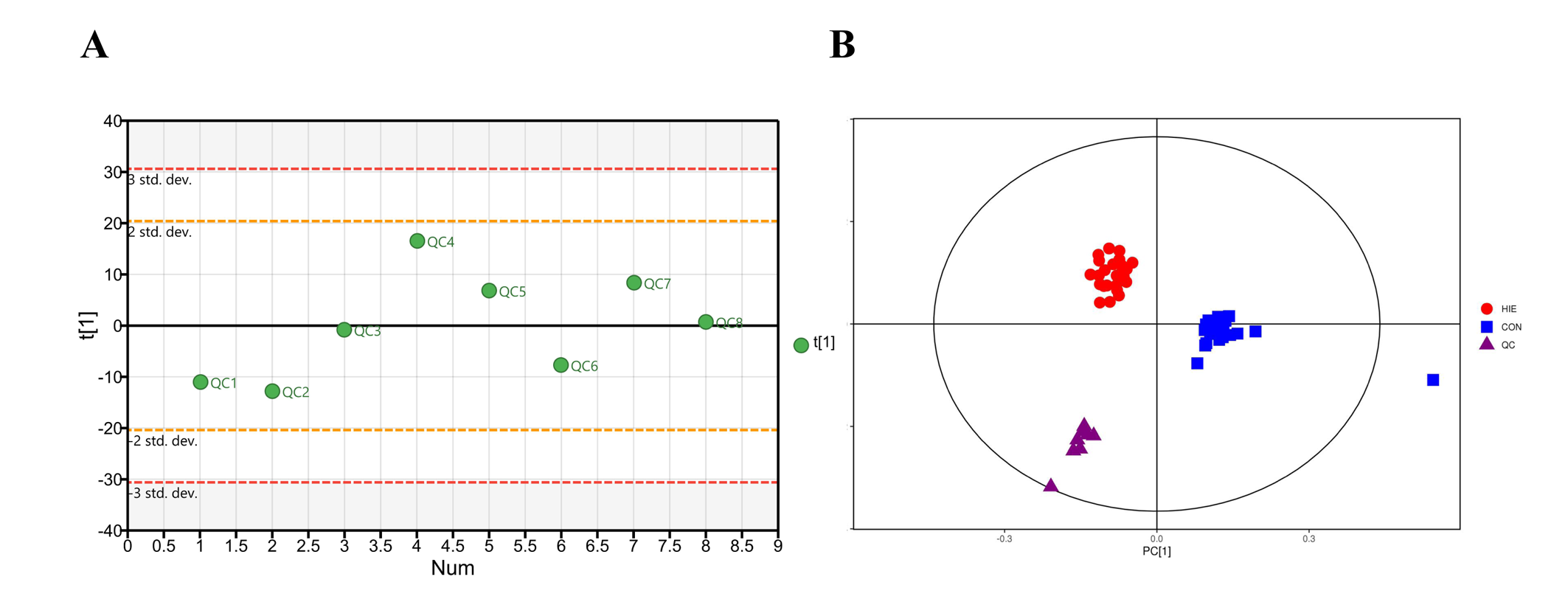
**Supplementary Figure 1 The quality control diagram.** A, One dimensional ; B, Two dimensional. HIE, hypoxic-ischaemic encephalopathy; CON, healthy group.


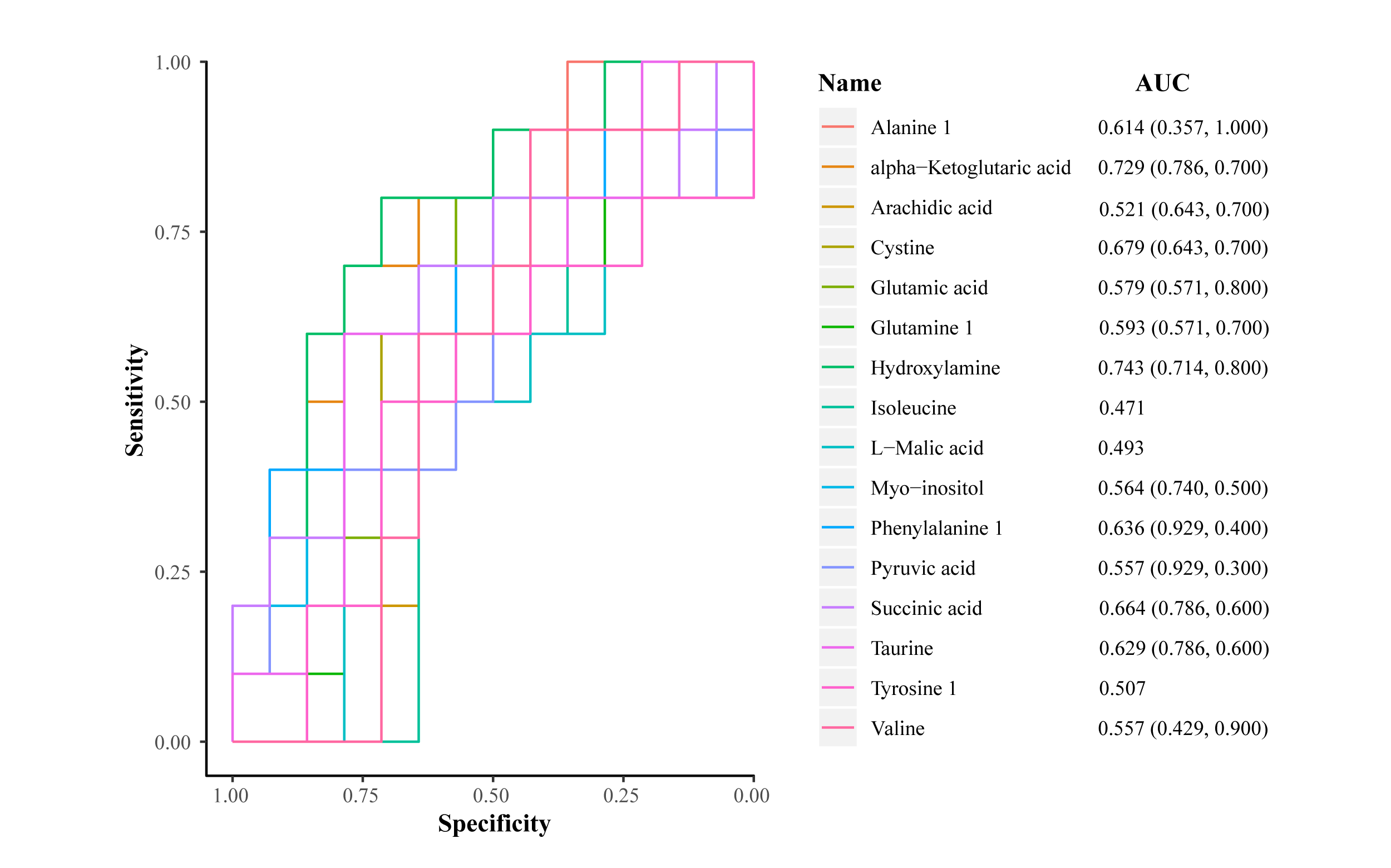
**Supplementary Figure 2 The prediction of diagnostic value of differential candidate metabolites on the severity of HIE.** AUC, area under the curve; ROC, receiver operating characteristic. HIE, hypoxic-ischaemic encephalopathy.


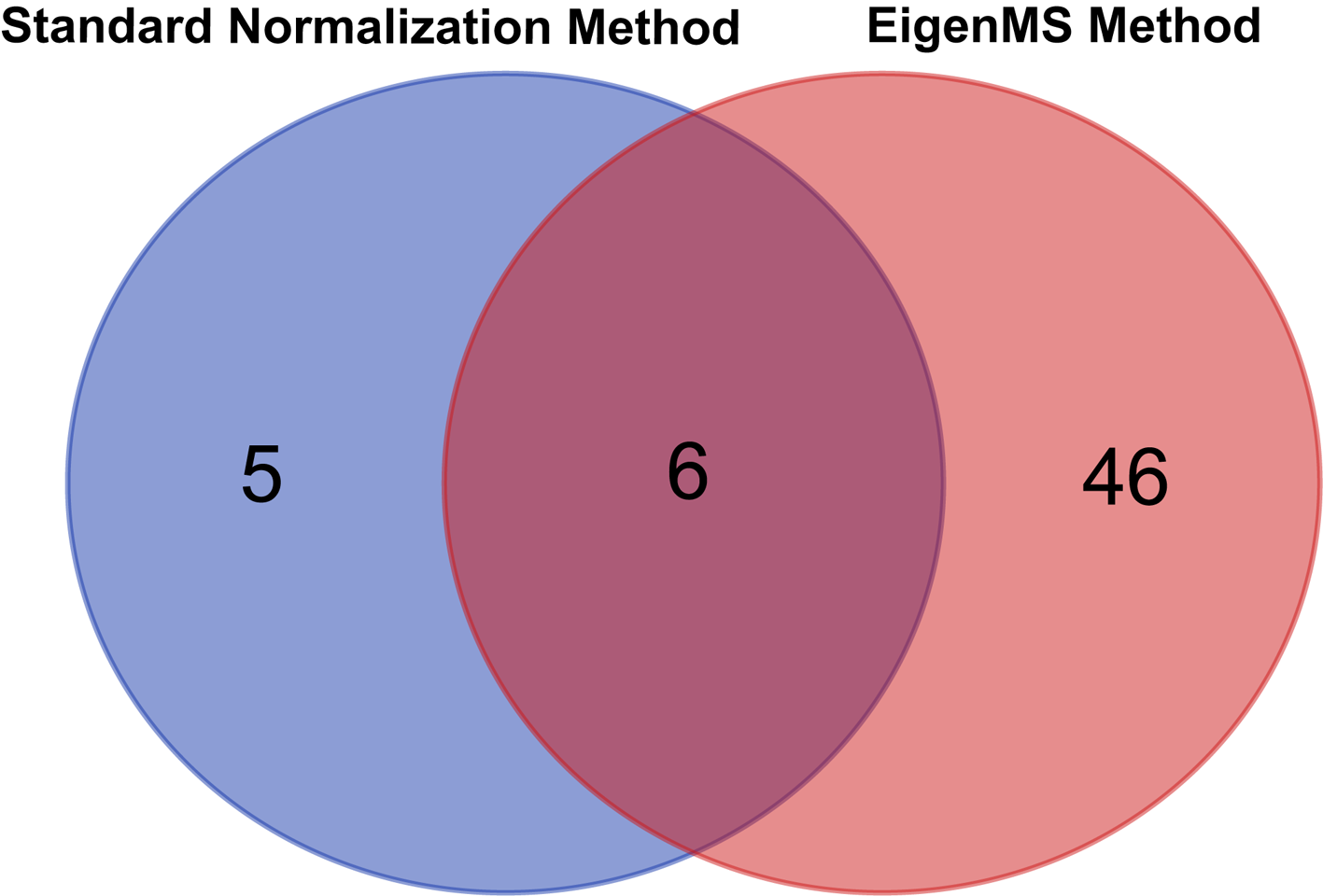


**Supplementary Figure 3 Comparison of differential metabolites screened by different data standardization methods.** A, Standard Normalized Method; B, EigenMS Method.
